# Cross-cultural adaptation of the Brazilian version of the Speech-Specific Reinvestment Scale – SSRS

**DOI:** 10.1590/2317-1782/20212020385

**Published:** 2022-01-21

**Authors:** Bruna Rainho Rocha, Andus Wing-Kuen Wong, Estella Pui-Man Ma, Mara Behlau

**Affiliations:** 1 Programa de Pós-graduação em Distúrbios da Comunicação Humana, Departamento de Fonoaudiologia, Universidade Federal de São Paulo – UNIFESP - São Paulo (SP), Brasil; 2 Nam Shan Psychology Laboratory, Department of Social and Behavioural Sciences, University of Hong Kong, Hong Kong, China; 3 Division of Speech and Hearing Sciences, University of Hong Kong, Hong Kong, China

**Keywords:** Voice, Validation Studies, Communication, Voice Quality, Speech, Language and Hearing Sciences, Questionnaires, Evaluation Studies, Translations

## Abstract

**Purpose:**

To present the cross-cultural equivalence of the Brazilian version of the Specific Reinvestment Scale in Speech – SRRS through its cultural and linguistic adaptation.

**Methods:**

After the SSRS was translated into Brazilian Portuguese, the back-translation was done and the items were compared. Discrepancies were modified by consensus of a committee of SLPs. The SSRS, named “Escala de Reinvestimento Específico na Fala – EREF”, has 39 questions and six alternatives in the answer key: “strongly disagree”, “disagree”, “slightly disagree”, “slightly agree”, “agree” and “strongly agree”. The mean score is computed by the sum of each subdimension. Negative items may not be included in the EREF scoring or need reversed coding process before using them. For cultural equivalence, the EREF was applied to a total of 74 professionals working in an activity involving communication with the public, speakers of Brazilian Portuguese as a first language, with an extra item in the answer key - “not applicable” - to identify issues that might not have been understood or were not appropriate for the target population and Brazilian culture.

**Results:**

The scale was initially applied to 56 participants, thirteen of whom found it difficult to complete 27 questions. After adaptation of those sentences, the modified EREF was applied to 13 more participants and no further cultural and / or conceptual barriers were found.

**Conclusion:**

Cultural equivalence between the SSRS and its translated version to Brazilian Portuguese – EREF was verified. The next steps for the EREF validation for Brazilian Portuguese will be carried out.

## INTRODUCTION

Communication is fundamental in all spheres of human relationships, whether in personal, business, or educational relationships. The term communicate comes from the Latin – *communicare* – which means “to make it common”^(1)^. In other words, communication is efficient when the interlocutor can transmit the desired message to the receiver, who fully understands it. The message can be encoded in different ways, whether verbal – through speech or writing – or non-verbal – such as using gestures and facial expressions. In an oral speech, verbal and non-verbal language is always present, whether the interlocutor is aware of them or not, and for good communication, one must agree with the other, completing each other and in coherence.

Communication has always been important and has stood out more and more since the professions that use communication as the main factor in the development of their work are increasing at the expense of manual work^(2)^. At the end of the 20^th^ century, 62% of the workforce based their livelihood on communication skills - hearing, voice, speech, and language - and 38% who did not use communication for work - such as farmers and workers – needed it for proper insertion into society. In other words, communication is fundamental for professional and personal growth, and its competence is increasingly demanded in society^(3)^.

Developing communication skills and bringing verbal and non-verbal language into agreement requires training and knowledge. To help those people with difficulty and make this training more specific and direct, it is important to assess the skills involved in communication, including self-assessment protocols.

Self-assessment protocols are important tools for measuring individuals' knowledge about the impact of their condition – whether it is a problem or something to be improved – on their social and professional relationships^(4)^. Despite their frequent use, most of them are still aimed at health issues such as quality of life questionnaires. In Brazil, there are still few instruments capable of investigating the self-assessment of an individual's communication. However, this panorama should change due to the importance of communication.

The “Speech-Specific Reinvestment Scale - SSRS”^(5)^ is a new psychometric measure that aims to quantify someone's predisposition to exercise conscious control and monitoring of speech. It encompasses issues not only of conscious control over speech movements (tongue, lip, and jaw movement) but also conscious monitoring of the content and manner of speaking, as well as facial and body movement to achieve the ultimate goal of communication, that is, to allow the recipient to perceive and understand the speech.

The scale was developed based on the reference of the Reinvestment Theory^(6-8)^ on body limb motor control. According to this theory, motor skills require two types of processing: implicit and explicit. The first is beneficial for well-practiced, relatively automated motor skills, working with pre-established knowledge and requiring little attention and working memory resources. On the other hand, the second is rule-based and requires considerable attention and working memory resources.

People can be high or low reinvestors and those who reinvest high are more likely to fail in stressful situations that involve complex skills and are linked to rules^(6-8)^. Thus, when elaborating the SSRS scale, the authors hypothesized that the personality trait predisposing to awareness of control and monitoring of speech would be negatively related to performance in conversational speech. The results pointed to the confirmation of the hypothesis.

Also, the results validated four SSRS sub-dimensions, one of control and three of speech monitoring: 1. Sub-dimension of self-awareness of speech movement - SASN, 2. Sub-dimension of public awareness of the way of speaking - PASC, 3. Sub-dimension of public awareness of movement during the speaking content - PMASC, 4. Sub-dimension of public awareness of movement during speech - PAMS^(5)^. The scale enables to assessment of the sub-dimensions distinctly or jointly, which makes it even more robust to examine any variable effects on speech performance, including non-verbal ones, as suggested by the Reinvestment Theory literature^(9)^.

Since the Speech-Specific Reinvestment Scale – SSRS^(5)^ was developed in English, to use it in other languages, it must be translated and culturally adapted according to the international rules of the Scientific Advisory Committee of Medical Outcome Trust^(10)^.

This study aimed to carry out the cultural equivalence of the Brazilian version of the SSRS scale, through its cultural and linguistic adaptation.

We should emphasize that, as this is a new scale, there is still no repercussion of its use, although it has great potential. The Brazilian adaptation is the first to be carried out with the instrument.

## METHODS

After formal authorization from the authors to use the Speech-Specific Reinvestment Scale - SSRS^(5)^ instrument, the research was approved by the Research Ethics Committee of the Federal University of São Paulo/CEP UNIFESP (opinion number 4.356.465 and CAAE: 36606720.0 .0000.5505). All participants signed the Informed Consent Form – ICF.

Adult professionals participated in an activity involving oral communication with the people, with a minimum experience of three months in the position. There was no distinction of the level of communication use, age, or socioeconomic-cultural level for this study.

The original version was translated into Brazilian Portuguese by two speech-language therapists fluent in the foreign language (Translator 1 and Translator 2). These translations were superimposed, resulting in the first version in Portuguese – PV, maintaining the conceptual integrity of the items. The back-translation was performed by a third speech-language therapist, also fluent in English, without access to the original version of the instrument and the study objectives.

The translation and back-translation were compared to each other and the original instrument. A committee composed of five speech-language therapists specialized in voice, with proficiency in English and knowledge of specific vocabulary for communication assessment analyzed and discussed the existing discrepancies. The necessary changes were carried out by consensus resulting in the Committee's version 1 – the semantic and language equivalence version, entitled *Escala de Reinvestimento Específico da Fala – EREF*.

The EREF followed the original protocol, remaining with 39 questions and six alternatives to mark the frequency of occurrence of the situation described in: totally disagree (1), disagree (2), slightly disagree (3), slightly agree (4), agree (5), totally agree (6). At that time, the four sub-dimensions of the scale were still maintained: 1. Sub-dimension of self-awareness of speech movement, 2. Sub-dimension of public awareness of the way of speaking, 3. Sub-dimension of public awareness of movement during the speaking content, 4. Sub-dimension of public awareness of movement during speech. The score for each sub-dimension of SSRS^(5)^ is calculated using the average scoring method. The average composite score for the EREF is calculated by the equally weighted sum of the four average scores for the sub-dimensions. To calculate the averages of each sub-dimension and composite average score, these steps are followed:

Q.1-13: Sub-dimension of self-awareness of speech movement

Total score: ___ ÷ 13 = Average sub-dimension score: ___

Q.15-27: Sub-dimension of public awareness of the way of speaking

Total score: ___ ÷ 13 = Average sub-dimension score:___

Q.29-33: Sub-dimension of public awareness of movement during the speaking content

Total Score: ___ ÷ 5 = Average sub-dimension score: ___

Q.35-38: Sub-dimension of public awareness of movement during speech

Total Score: ___ ÷ 4 = Average sub-dimension score: ___

Total = Sum of average scores:_________

It is important to emphasize that there are four negative questions in the EREF - one for each subdimension of the scale. Questions 14, 28, 34, and 39 are not part of the total EREF score, as they were developed to check the quality of individuals' responses, including reliability. If the evaluator feels the need to include them in the score, he must remember to perform the reverse score, as described in the original protocol^(5)^.

For cultural equivalence, the EREF was applied to 74 speaking voice professionals with the addition of the option “not applicable” to identify issues not understood or inappropriate for the target population and Brazilian culture. A space for filling in observations was also added at the end of the scale, allowing volunteers to explain their difficulties or doubts. If volunteers found any difficulty in answering the scale, a new translation and adaptation should be carried out until cultural and/or conceptual barriers were no longer found.

## RESULTS

In the first stage, 61 individuals responded to the EREF online. Five answers were incompletes. Therefore, 56 individuals were included in the sample. Of these, 13 indicated “not applicable” in at least one of the scale's questions. A total of 27 questions were identified as not understood or not appropriate for the target population and Brazilian culture and were discussed by the Speech-Language Pathologist committee. Question two, for example, was changed from “I am aware of how my mouth moves when I speak” to “I notice how my mouth moves when I speak” to use an easily accessible language and closer to all respondents. Also, it was decided to add the pronoun “I” to all questions, excluding sentences with hidden subjects to avoid any misinterpretation. As an example, we have question five, in which the phrase “I think of the movement of my lips when speaking” was adjusted to “I think of the movement of my lips when I speak”.

Also, the Committee of Speech-Language Pathologists analyzed what the respondents had written in the space for observations, and they concluded that many had marked “not applicable” not because the questions had not been understood, but because those individuals had never paid attention to the context of the question. Thus, they should have marked “disagree” as they do not agree with that statement instead of “not applicable”. Therefore, the instructions for filling out the scale were revised for proper filling.

The initial instruction was: “Select the most appropriate options to indicate how much you agree with each of the statements: (1) Totally Disagree, (2) Disagree, (3) Slightly Disagree, (4) Slightly Agree, (5) Agree, (6) Totally agree (Note: There is no right or wrong answer for each sentence). If you do not understand any of the phrases or understand that it is not appropriate for the scale, select the option “not applicable”. At the end of the sentences, there is a space to fill in “comments.” After deliberation by the Committee of Speech-Language Pathologists and the understanding that the instructions should be clearer, there was a change to: “Select the most appropriate options to indicate how much you agree with each of the statements: (1) Totally disagree, (2) Disagree, (3) Slightly disagree, (4) Slightly agree, (5) Agree, (6) Totally agree (Note: There is no right or wrong answer for each sentence). If you do not understand any of the phrases or understand that this question within the questionnaire does not make sense, select the option “not applicable”. This marking should only be performed in these cases. If you select “not applicable”, we ask that you explain your difficulties or doubts in the space dedicated to filling in the comments at the end of the sentences.”

**Figure 1:**
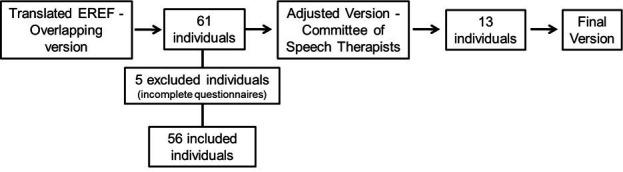
Organizational chart for completing the survey questionnaire and inclusion and exclusion of participants

The adjusted EREF - version 2 of the Speech-Language Pathologist Committee - was applied to 13 new respondents, totaling 69 volunteers included in the sample and no cultural and/or conceptual barriers were identified. Figure 1 shows the mapping of the participants and Chart 1 shows their characterization.

**Chart 1:** Characterization of the sample according to gender, age (in years), and profession.

Variables	N	%
**Gender**		
**Female**	**45**	**65.2**
**Male**	**24**	**34.8**
		
**Age**		
**18** |–|**30**	**27**	**39.1**
**31** |–| **45**	**16**	**23.2**
**46** |–| **60**	**20**	**29**
**60** |–| **86**	**6**	**8.7**
		
**Profession**		
**Professor(a)**	**9**	**13**
**Administrator**	**6**	**8.7**
**Entrepreneur**	**6**	**8.7**
**Speech-language therapist**	**5**	**7.2**
**Publicist**	**5**	**7.2**
**Engineer**	**4**	**5.9**
**Lawyer**	**3**	**4.4**
**Retired**	**3**	**4.4**
**Businessperson**	**3**	**4.4**
**Student**	**3**	**4.4**
**Analyst**	**2**	**2.9**
**Architect**	**2**	**2.9**
**Consultant**	**2**	**2.9**
**Cook**	**2**	**2.9**
**Manager**	**2**	**2.9**
**Journalist**	**2**	**2.9**
**Doctor**	**2**	**2.9**
**Public relations**	**2**	**2.9**
**Occupational therapist**	**2**	**2.9**
**Singer**	**1**	**1.4**
**Dentist**	**1**	**1.4**
**Writer**	**1**	**1.4**
**Promoter of Justice**	**1**	**1.4**

This last version applied, then resulted in the final version of the EREF, following cultural and linguistic equivalence. Chart 2 shows the entire process of translation, semantic and cultural equivalence of the SSRS into Brazilian Portuguese.

**Chart 2:** Process of translation and cultural adaptation of the Speech-Specific Reinvestment Scale - SSRS to Brazilian Portuguese

Questions	Original version in English	Translation 1 English-Portuguese	Translation 2 English-Portuguese	Superimposed version (PV)	Back translation of PV	Version 1 of the Committee	Final Version of the Committee
1	I examine my mouth movement when I speak.	Eu **analiso** o movimento da boca quando falo.	Eu **avalio/examino meu** movimento de boca quando eu falo.	Eu avalio meu movimento da boca quando eu falo.	When speaking I monitor my mouth movement	Eu avalio movimento de boca quando eu falo.	Eu avalio **O** movimento **DA MINHA BOCA** quando eu falo.
2	I am aware of the way my mouth moves when I speak.	Eu estou ciente de como minha boca se move quando falo.	Estou ciente da como minha boca se move quando falo.	Eu estou ciente de como minha boca se move quando eu falo.	I am aware of how my mouth moves when speaking	Eu estou ciente de como minha boca se move quando eu falo.	Eu **PERCEBO** como **A** minha boca se move quando eu falo.
3	I take time to reflect on my mouth movement after speaking.	Eu dedico tempo para refletir sobre o movimento da minha boca depois de falar.	Eu dedico tempo para refletir sobre o movimento da minha boca depois de falar.	Eu dedico tempo para refletir sobre o movimento da minha boca depois de falar.	After speaking, I think about my mouth movement	Eu dedico tempo para refletir sobre o movimento da minha boca depois de falar.	Eu dedico tempo para refletir sobre o movimento da minha boca depois de falar.
4	I take time to think about my mouth movement before speaking.	Eu dedico tempo para pensar no movimento da minha boca antes de falar.	Eu dedico tempo para pensar no movimento da minha boca antes de falar.	Eu dedico tempo para pensar no movimento da minha boca antes de falar.	Before speaking, I think about my mouth movement	Eu dedico tempo para pensar no movimento da minha boca antes de falar.	Eu dedico tempo para pensar no movimento da minha boca antes de falar.
5	I think about the movement of my lips when I speak.	Eu penso no movimento dos meus lábios quando falo.	Eu penso no movimento dos meus lábios quando falo.	Eu penso no movimento dos meus lábios quando falo.	I think about my lips movements when speaking	Eu penso no movimento dos meus lábios quando falo.	Eu penso no movimento dos meus lábios quando **EU** falo.
6	I think about the movement of my tongue when I speak.	Eu penso no movimento da minha língua quando falo.	Eu penso no movimento da minha língua quando falo.	Eu penso no movimento da minha língua quando falo.	I think about my toungue movement when speaking	Eu penso no movimento da minha língua quando falo.	Eu penso no movimento da minha língua quando **EU** falo.
7	I think about the movement of my jaw when I speak.	Eu penso no movimento da minha mandíbula quando falo.	Eu penso no movimento da minha mandíbula quando falo.	Eu penso no movimento da minha mandíbula quando falo.	I think about my jaw movement when speaking	Eu penso no movimento da minha mandíbula quando falo.	Eu penso no movimento da minha mandíbula quando **EU** falo.
8	I think about the movement of my larynx when I speak.	Eu penso no movimento da minha laringe quando falo.	Eu penso no movimento da minha laringe quando eu falo.	Eu penso no movimento da minha laringe quando falo.	I think about my larynx movement when speaking	Eu penso no movimento da minha laringe quando falo.	Eu penso no movimento da minha laringe quando **EU** falo.
9	I try to figure out how my mouth movement generates pronunciation.	Eu tento descobrir como o movimento da minha boca gera a pronúncia **DAS PALAVRAS.**	Eu tento descobrir como o movimento da minha boca gera a pronúncia.	Eu tento descobrir como o movimento da minha boca gera a pronúncia das palavras.	I try figuring out how my mouth moments produce words	Eu tento descobrir como o movimento da minha boca gera a pronúncia das palavras.	Eu tento descobrir como o movimento da minha boca gera a pronúncia das palavras.
10	I rethink how my mouth movement has helped me speak smoothly.	Eu repenso em **COMO O MOVIMENTO DA MINHA BOCA ME AJUDOU A FALAR SEM DIFICULDADES.**	Eu repenso em como o movimento da minha boca me ajudou a falar **SUAVEMENTE/SEM DIFICULDADES.**	Eu repenso em como o movimento da minha boca me ajudou a falar sem dificuldades.	I overthink how my mouth movement helped me speak easily	Eu repenso em como o movimento da minha boca me ajudou a falar sem dificuldades.	Eu repenso em como o movimento da minha boca me ajudou a falar sem dificuldades.
11	I think about how my mouth movement has helped me speak smoothly.	Eu penso em como o movimento da minha boca me ajudou a falar sem dificuldades.	Penso em como o movimento da minha boca me ajudou a falar **SUAVEMENTE**/sem dificuldades.	Eu penso em como o movimento da minha boca me ajudou a falar sem dificuldades.	I think how my mouth movement helped me speak easily	Eu penso em como o movimento da minha boca me ajudou a falar sem dificuldades.	Eu penso em como o movimento da minha boca me ajudou a falar sem dificuldades.
12	I try to understand how my speech arises from mouth movement.	Eu tento entender como minha fala surge do movimento **DA MINHA** boca.	Eu tento entender como minha fala surge do movimento da boca.	Eu tento entender como minha fala surge do movimento da minha boca.	I try to understand how my speech is produced from my mouth movement	Eu tento entender como minha fala surge do movimento da minha boca.	Eu tento entender como minha fala surge do movimento da minha boca.
13	I am aware of how my mouth moves for what I want to speak.	**EU** estou ciente de como minha boca se move para o que eu quero falar.	Estou ciente de como minha boca se move para o que eu quero falar.	Eu estou ciente de como minha boca se move para o que eu quero falar.	I am aware of how my mouth moves when speaking what I want	Eu estou ciente de como minha boca se move para o que eu quero falar.	Eu **PERCEBO** como minha boca se move para o que eu quero falar.
14	I do not try to figure out how my mouth movement generates pronunciation.	**EU** não tento descobrir como o movimento da minha boca gera **A PRONÚNCIA DAS PALAVRAS**.	Não tento descobrir como o movimento da minha boca gera pronúncia.	Eu não tento descobrir como o movimento da minha boca gera a pronúncia das palavras.	I do not try figuring out how my mouth moments produce words	Eu não tento descobrir como o movimento da minha boca gera a pronúncia das palavras.	Eu não tento descobrir como o movimento da minha boca gera a pronúncia das palavras.
15	I care about the voice being used to present myself to others.	Eu me preocupo com a minha voz **QUANDO EU ME APRESENTO** aos outros.	Eu me preocupo **COM A VOZ SENDO USADA** para me apresentar aos outros.	Eu me preocupo com a voz que eu uso para para me apresentar aos outros.	My voice concerns me when I introduce myself	Eu me preocupo com a voz que eu uso para para me apresentar aos outros.	Eu me preocupo com a voz que eu uso para me apresentar aos outros.
16	Before I talk to others, I think about how my pronunciation is.	Antes de falar com outras pessoas, **EU** penso em como está minha pronúncia.	Antes de falar com outras pessoas, penso em como está a minha pronúncia.	Antes de falar com outras pessoas, eu penso em como está minha pronúncia.	Before speaking with other people I think about my pronunciation	Antes de falar com outras pessoas, eu penso em como está minha pronúncia.	Antes de falar com outras pessoas, eu penso como está minha pronúncia.
17	I care about my pronunciation when I am invited to express my views.	Eu me preocupo com a minha pronúncia quando sou convidado a expressar meus pontos de vista.	Eu me preocupo com a minha pronúncia quando sou convidado a expressar meus pontos de vista.	Eu me preocupo com a minha pronúncia quando sou convidado a dar minhas opiniões.	My pronunciation concerns me when I am invited to give opinions	Eu me preocupo com a minha pronúncia quando sou convidado a dar minhas opiniões.	Eu me preocupo com a minha pronúncia quando **EU** sou convidado a dar minhas opiniões.
18	I am concerned about what other people think about my volume of speech.	Eu fico preocupado com o que as outras pessoas pensam sobre o volume **DE MINHA VOZ.**	Estou preocupado com o que as outras pessoas pensam sobre o meu volume **DE FALA.**	Estou preocupado com o que as outras pessoas pensam sobre o volume da minha fala.	I am concern of what people think about my speech loudness	Estou preocupado com o que as outras pessoas pensam sobre o volume da minha fala.	**EU FICO PREOCUPADO(A)** com o que as outras pessoas pensam sobre o volume da minha fala.
19	I care about my volume of speech when I am presenting myself to others.	Eu me preocupo com o **VOLUME DE MINHA VOZ** quando eu **ME APRESENTO** a outras pessoas.	Eu me preocupo com o **MEU VOLUME DE FALA** quando **ESTOU ME APRESENTANDO** para os outros.	Eu me preocupo com o volume da minha fala quando estou me apresentando a outras pessoas.	My speech loudness concerns me when I introduce myself	Eu me preocupo com o volume da minha fala quando estou me apresentando a outras pessoas.	Eu me preocupo com o volume da minha fala quando **EU** estou me apresentando **PARA** outras pessoas.
20	I worry about making a good impression with my volume of speech.	Eu me preocupo em causar uma boa impressão com o **VOLUME DE MINHA VOZ.**	Eu me preocupo em causar uma boa impressão no meu **VOLUME DE FALA**.	Eu me preocupo em causar uma boa impressão no volume da minha fala.	I am concern with making a good impression on my speech loudness	Eu me preocupo em causar uma boa impressão no volume da minha fala.	Eu me preocupo em causar uma boa impressão no volume da minha fala.
21	Before I talk to others, I think about my volume of speech.	Antes de falar com outras pessoas, eu penso no **VOLUME DA MINHA VOZ.**	Antes de falar com outras pessoas, penso no meu **VOLUME DE FALA**.	Antes de falar com outras pessoas, eu penso no volume da minha fala.	Before speaking with other people I think about my speech loudness	Antes de falar com outras pessoas, eu penso no volume da minha fala.	Antes de falar com outras pessoas, eu penso no volume da minha fala.
22	I care about my volume of speech when I am invited to express my view.	Eu me preocupo com o **VOLUME DE MINHA VOZ** quando sou convidado a expressar minha opinião.	Eu me preocupo com meu **VOLUME DE FALA** quando sou convidado a expressar minha opinião.	Eu me preocupo com o volume da minha fala quando sou convidado a dar minha opinião.	I am concern with my speech loudness when I am asked to give my opinion	Eu me preocupo com o volume da minha fala quando sou convidado a dar minha opinião.	Eu me preocupo com o volume da minha fala quando **EU** sou convidado(a) a dar minha opinião.
23	I am concerned about what other people think about my speech rate.	**EU FICO PREOCUPADO** com o que as outras pessoas pensam sobre a minha velocidade de fala.	**ESTOU PREOCUPADO** com o que as outras pessoas pensam sobre a minha velocidade de fala.	Eu fico preocupado com o que as outras pessoas pensam sobre a velocidade da minha fala.	I am concern of what people think about my speech rate	Eu fico preocupado com o que as outras pessoas pensam sobre a velocidade da minha fala.	Eu fico preocupado(a) com o que as outras pessoas pensam sobre a velocidade da minha fala.
24	I care about my speech rate when I am presenting myself to others.	Eu me preocupo com a velocidade de minha fala quando estou me apresentando a outras pessoas.	Eu me preocupo com a minha velocidade de fala quando estou me apresentando para outras pessoas.	Eu me preocupo com a velocidade da minha fala quando estou me apresentando a outras pessoas.	My speech rate concerns me when I introduce myself	Eu me preocupo com a velocidade da minha fala quando estou me apresentando a outras pessoas.	Eu me preocupo com a velocidade da minha fala quando **EU** estou me apresentando **PARA** outras pessoas.
25	I worry about making a good impression with my speech rate.	Eu me preocupo em causar uma boa impressão com a **VELOCIDADE DE MINHA FALA**.	Eu me preocupo em causar uma boa impressão com **A MINHA VELOCIDADE DE FALA**.	Eu me preocupo em causar uma boa impressão com a velocidade da minha fala.	I am concern with making a good impression on my speech rate	Eu me preocupo em causar uma boa impressão com a velocidade da minha fala.	Eu me preocupo em causar uma boa impressão com a velocidade da minha fala.
26	Before I talk to others, I think about my speech rate.	Antes de falar com outras pessoas, eu penso na **VELOCIDADE DE MINHA FALA.**	Antes de falar com outras pessoas, eu penso **NA MINHA VELOCIDADE DE FALA.**	Antes de falar com outras pessoas, eu penso na velocidade da minha fala.	Before speaking with other people I think about my speech rate	Antes de falar com outras pessoas, eu penso na velocidade da minha fala.	Antes de falar com outras pessoas, eu penso na velocidade da minha fala.
27	I care about my speech rate when I am invited to express my view.	Eu me preocupo com a **VELOCIDADE DA MINHA FALA** quando sou chamado a expressar minha opinião.	Eu me preocupo com a **MINHA VELOCIDADE DE FALA** quando sou convidado a expressar minha opinião.	Eu me preocupo com a velocidade da minha fala quando sou convidado a dar minha opinião.	I am concern with my speech rate when I am asked to give my opinion	Eu me preocupo com a velocidade da minha fala quando sou convidado a dar minha opinião.	Eu me preocupo com a velocidade da minha fala quando **EU** sou convidado a dar minha opinião.
28	Before I talk to others, I do not think about how my pronunciation is.	Antes de falar com outras pessoas, eu não penso em como está minha pronúncia **DAS PALAVRAS**.	Antes de falar com outras pessoas, não penso em como está minha pronúncia.	Antes de falar com outras pessoas, eu não penso em como está minha pronúncia das palavras.	Before speaking with other people I do not think about my pronunciation	Antes de falar com outras pessoas, eu não penso em como está minha pronúncia das palavras.	Antes de falar com outras pessoas, eu não penso em como está minha pronúncia das palavras.
29	I am concerned about what other people think about my speech content.	**EU FICO PREOCUPADO** com o que as outras pessoas pensam **SOBRE O CONTEÚDO DE MINHA FALA**.	**ESTOU PREOCUPADO** com o que as outras pessoas pensam **SOBRE O MEU CONTEÚDO DE FALA.**	Eu fico preocupado com o que as outras pessoas pensam sobre o conteúdo da minha fala.	The content of my speech concerns me when talking with other people	Eu fico preocupado com o que as outras pessoas pensam sobre o conteúdo da minha fala.	Eu fico preocupado com o que as outras pessoas pensam sobre o conteúdo da minha fala.
30	I care about how the content is organized when presenting myself to others.	Eu me preocupo com a organização do **CONTEÚDO DO QUE VOU FALAR** ao me apresentar a outras pessoas.	Eu me preocupo com a organização do conteúdo ao me apresentar a outras pessoas.	Eu me preocupo com a organização do conteúdo do que vou falar ao me apresentar a outras pessoas.	The content organization concerns me when I introduce myself	Eu me preocupo com a organização do conteúdo do que vou falar ao me apresentar a outras pessoas.	Eu me preocupo com a organização do conteúdo do que **EU** vou falar ao me apresentar **PARA** outras pessoas.
31	I worry about making a good impression with my speech content.	Eu me preocupo em causar uma boa impressão com o **CONTEÚDO DE MINHA FALA.**	Eu me preocupo em causar uma boa impressão com o **MEU CONTEÚDO DE FALA**.	Eu me preocupo em causar uma boa impressão com o conteúdo da minha fala.	I am concern with making a good impression with my speech content	Eu me preocupo em causar uma boa impressão com o conteúdo da minha fala.	Eu me preocupo em causar uma boa impressão com o conteúdo da minha fala.
32	Before I talk to others, I think about how the content of my speech is.	Antes de falar com outras pessoas, eu penso no **CONTEÚDO DO QUE VOU FALAR.**	Antes de falar com outras pessoas, penso **EM COMO ESTÁ O CONTEÚDO DO MEU DISCURSO.**	Antes de falar com outras pessoas, eu penso no conteúdo do que eu vou falar.	Before speaking with other people I think about my speech content	Antes de falar com outras pessoas, eu penso no conteúdo do que eu vou falar.	Antes de falar com outras pessoas, eu penso no conteúdo do que eu vou falar.
33	I care about my speech content when I am invited to express my views.	Eu me preocupo com o **CONTEÚDO DO QUE VOU FALAR QUANDO SOU CONVIDADO A DAR MINHAS OPINIÕES.**	Eu me preocupo com o **CONTEÚDO DA MINHA FALA QUANDO SOU CONVIDADO A EXPRESSAR MINHAS OPINIÕES.**	Eu me preocupo com o conteúdo do que vou falar quando sou convidado a dar minhas opiniões.	I am concern with my speech content when I am asked to give my opinion	Eu me preocupo com o conteúdo do que vou falar quando sou convidado a dar minhas opiniões.	Eu me preocupo com o conteúdo do que **EU** vou falar quando **EU** sou convidado(a) a dar minhas opiniões.
34	I am not concerned about what other people think about my speech content.	**EU NÃO FICO PREOCUPADO** com o que as outras pessoas pensam sobre o **CONTEÚDO DO QUE VOU FALAR**.	**NÃO ESTOU PREOCUPADO** com o que as outras pessoas pensam sobre o **CONTEÚDO DA MINHA FALA.**	Eu não fico preocupado com o que as outras pessoas pensam sobre o conteúdo do que eu vou falar.	I am not concern with what people think about my speech content	Eu não fico preocupado com o que as outras pessoas pensam sobre o conteúdo do que eu vou falar.	Eu não fico preocupado(a) com o que as outras pessoas pensam sobre o conteúdo do que eu vou falar.
35	I am concerned about what other people think about my facial movement when I speak.	**EU FICO PREOCUPADO** com o que as outras pessoas pensam sobre o **MOVIMENTO DE MINHA FACE** quando falo.	**ESTOU PREOCUPADO** com o que as outras pessoas pensam sobre o meu **MOVIMENTO FACIAL** quando falo.	Eu fico preocupado com o que as outras pessoas pensam sobre o movimento da minha face quando falo.	What people think about my face movements concerns me when speaking	Eu fico preocupado com o que as outras pessoas pensam sobre o movimento da minha face quando falo.	Eu fico preocupado(a) com o que as outras pessoas pensam sobre o movimento da minha face quando **EU** falo.
36	I am concerned about what other people think about my body movement when I speak.	**EU FICO PREOCUPADO** com o que as outras pessoas pensam sobre o movimento do meu corpo quando falo.	**ESTOU PREOCUPADO** com o que as outras pessoas pensam sobre o movimento do meu corpo quando falo.	Eu fico preocupado com o que as outras pessoas pensam sobre o movimento do meu corpo quando falo.	What people think about my body movements concerns me when speaking	Eu fico preocupado com o que as outras pessoas pensam sobre o movimento do meu corpo quando falo.	Eu fico preocupado(a) com o que as outras pessoas pensam sobre o movimento do meu corpo quando **EU** falo.
37	I worry about making a good impression with my facial movement when I speak.	Eu me preocupo em causar uma boa impressão com o **MOVIMENTO DE MINHA FACE** quando falo.	Eu me preocupo em causar uma boa impressão com **A MINHA MOVIMENTAÇÃO FACIAL** quando falo.	Eu me preocupo em causar uma boa impressão com o movimento da minha face quando falo.	I am concern with making a good impression regarding my face movements when I speak	Eu me preocupo em causar uma boa impressão com o movimento da minha face quando falo.	Eu me preocupo em causar uma boa impressão com o movimento da minha face quando **EU** falo.
38	I care about my facial movement when I am invited to express my views.	Eu me preocupo com o **MOVIMENTO DE MINHA FACE** quando sou **CHAMADO** a expressar **MEUS PONTOS DE VISTA.**	Eu me preocupo com **A MINHA MOVIMENTAÇÃO FACIAL** quando sou **CONVIDADO** a expressar **MINHAS OPINIÕES.**	Eu me preocupo com o movimento da minha face quando sou convidado a dar minhas opiniões.	I am concern with my face movement when I am asked to give my opinion	Eu me preocupo com o movimento da minha face quando sou convidado a dar minhas opiniões.	Eu me preocupo com o movimento da minha face quando **EU** sou convidado(a) a dar minhas opiniões.
39	I do not worry about making a good impression with my facial movement when I speak.	Eu não me preocupo em causar uma boa impressão com os **MOVIMENTOS DE MINHA FACE** quando falo.	Não me preocupo em causar uma boa impressão com a minha **MOVIMENTAÇÃO FACIAL** quando falo.	Eu não me preocupo em causar uma boa impressão com os movimentos da minha face quando falo.	I am not concern with making a good impression regarding my face movements when I speak	Eu não me preocupo em causar uma boa impressão com os movimentos da minha face quando falo.	Eu não me preocupo em causar uma boa impressão com os movimentos da minha face quando **EU** falo.

Source: elaborated by the authors

The final composition of the translated and the culturally adapted Brazilian version of the SSRS, called EREF (Annex 1), has 39 items, like the original protocol.

## DISCUSSION

Communication plays a great power within today's society. Using communication, we can convince, persuade, influence, arouse interests and feelings, and even provoke expectations in the interlocutor^(11)^. Within an organization, for example, well-used communication can establish peaceful relationships, homogenization, and integration of ideas. Communication is a means as well as a tool and, considering its fundamental role, there must be a way to evaluate it, in its different aspects. For this purpose, the SSRS was created.

When a new protocol is proposed, there are rules in its development so that there are no difficulties in its application with the target population. The same must be thought for the use of this protocol in other countries. Therefore, the validation of the protocol for each language and culture is essential. Obtaining cultural equivalence is the first step for the validation of protocols and aims to eliminate cultural and linguistic barriers between the instrument and its target population in different countries^(10)^. The cultural equivalence model used for this research has already been successfully performed in validations of other protocols in Brazil^(12-15)^.

The cultural equivalence of the SSRS began with the cultural and linguistic adaptation, in which the version of the scale for semantic and language equivalence is applied. In this phase, 27 questions were identified as not understood or not appropriate for the target population and Brazilian culture and were discussed by the Speech-Language Pathologist Committee. The high number of questions with barriers for the target population (69%) raised the discussion by the Speech-Language Pathologists Committee regarding the understanding of the scale by the target population. Many respondents had not understood the real function of the “not applicable” option and instead of ticking it when they believed the question was not appropriate to the scale, some were checking this option because they did not fulfill the context of the question in their tasks of communication. That is, instead of answering the question following the frequency of responses from 1 to 6, the respondents put “not applicable”. This situation led to the need to review the instructions for filling out the scale and, in a new application, no difficulties were observed.

The cultural adaptation step is essential for the scale's language to approach the target population and for the consistency of the form of communication to be maintained in all questions. This step should be performed as many times as necessary until the scale/protocol is accepted by the target population. The SRSS for Brazilian Portuguese needed two reviews to be accepted.

With the completion of the cultural and linguistic equivalence process for Brazilian Portuguese, the EREF validation process that is the name of the adapted scale will begin. Its use will be important, as most voice self-assessment protocols are disease-specific, are not suitable for individuals with healthy voices, and do not address other oral communication issues. The SSRS is a specific scale for voice professionals and will be essential in Brazil to assess those who use the voice as their work tool, regardless of the level of classification of professional voice use.

## CONCLUSION

Cultural equivalence between the SSRS and its version translated into Brazilian Portuguese called the EREF was verified. Therefore, the other validation of the EREF for Brazilian Portuguese will be started.

## Annex 1. **Translated and culturally adapted version of the Speech-Specific Reinvestment Scale –SSRS, called *Escala de Reinvestimento Específico da Fala* – EREF in Portuguese.**

Pergunta 1 a 39: As frases a seguir descrevem sua autorreflexão enquanto fala no português brasileiro.

As questões precisam ser lidas com atenção. Algumas são positivas e outras negativas.

Selecione as opções mais adequadas para indicar o quanto você concorda com cada uma das questões:

1: Discordo totalmente

2: Discordo

3: Discordo ligeiramente

4: Concordo ligeiramente

5: Concordo

6: Concordo totalmente

(Nota: Não há resposta certa ou errada para cada frase)

**Table:**

		**Discordo Totalmente**			**Concordo Totalmente**		
**1**	**Eu avalio o movimento da minha boca quando eu falo.**	**1**	**2**	**3**	**4**	**5**	**6**
**2**	**Eu percebo como a minha boca se move quando eu falo.**	**1**	**2**	**3**	**4**	**5**	**6**
**3**	**Eu dedico tempo para refletir sobre o movimento da minha boca depois de falar.**	**1**	**2**	**3**	**4**	**5**	**6**
**4**	**Eu dedico tempo para pensar no movimento da minha boca antes de falar.**	**1**	**2**	**3**	**4**	**5**	**6**
**5**	**Eu penso no movimento dos meus lábios quando eu falo.**	**1**	**2**	**3**	**4**	**5**	**6**
**6**	**Eu penso no movimento da minha língua quando eu falo.**	**1**	**2**	**3**	**4**	**5**	**6**
**7**	**Eu penso no movimento da minha mandíbula quando eu falo.**	**1**	**2**	**3**	**4**	**5**	**6**
**8**	**Eu penso no movimento da minha laringe quando eu falo.**	**1**	**2**	**3**	**4**	**5**	**6**
**9**	**Eu tento descobrir como o movimento da minha boca gera a pronúncia das palavras.**	**1**	**2**	**3**	**4**	**5**	**6**
**10**	**Eu repenso em como o movimento da minha boca me ajudou a falar sem dificuldades.**	**1**	**2**	**3**	**4**	**5**	**6**
**11**	**Eu penso em como o movimento da minha boca me ajudou a falar sem dificuldades.**	**1**	**2**	**3**	**4**	**5**	**6**
**12**	**Eu tento entender como minha fala surge do movimento da minha boca.**	**1**	**2**	**3**	**4**	**5**	**6**
**13**	**Eu percebo como minha boca se move para o que eu quero falar.**	**1**	**2**	**3**	**4**	**5**	**6**
**14**	**Eu não tento descobrir como o movimento da minha boca gera a pronúncia das palavras.**	**1**	**2**	**3**	**4**	**5**	**6**
**15**	**Eu me preocupo com a voz que eu uso para me apresentar aos outros.**	**1**	**2**	**3**	**4**	**5**	**6**
**16**	**Antes de falar com outras pessoas, eu penso como está minha pronúncia.**	**1**	**2**	**3**	**4**	**5**	**6**
**17**	**Eu me preocupo com a minha pronúncia quando eu sou convidado a dar minhas opiniões.**	**1**	**2**	**3**	**4**	**5**	**6**
**18**	**Eu fico preocupado(a) com o que as outras pessoas pensam sobre o volume da minha fala.**	**1**	**2**	**3**	**4**	**5**	**6**
**19**	**Eu me preocupo com o volume da minha fala quando eu estou me apresentando para outras pessoas.**	**1**	**2**	**3**	**4**	**5**	**6**
**20**	**Eu me preocupo em causar uma boa impressão no volume da minha fala.**	**1**	**2**	**3**	**4**	**5**	**6**
**21**	**Antes de falar com outras pessoas, eu penso no volume da minha fala.**	**1**	**2**	**3**	**4**	**5**	**6**
**22**	**Eu me preocupo com o volume da minha fala quando eu sou convidado(a) a dar minha opinião.**	**1**	**2**	**3**	**4**	**5**	**6**
**23**	**Eu fico preocupado(a) com o que as outras pessoas pensam sobre a velocidade da minha fala.**	**1**	**2**	**3**	**4**	**5**	**6**
**24**	**Eu me preocupo com a velocidade da minha fala quando eu estou me apresentando para outras pessoas.**	**1**	**2**	**3**	**4**	**5**	**6**
**25**	**Eu me preocupo em causar uma boa impressão com a velocidade da minha fala.**	**1**	**2**	**3**	**4**	**5**	**6**
**26**	**Antes de falar com outras pessoas, eu penso na velocidade da minha fala.**	**1**	**2**	**3**	**4**	**5**	**6**
**27**	**Eu me preocupo com a velocidade da minha fala quando eu sou convidado a dar minha opinião.**	**1**	**2**	**3**	**4**	**5**	**6**
**28**	**Antes de falar com outras pessoas, eu não penso em como está minha pronúncia das palavras.**	**1**	**2**	**3**	**4**	**5**	**6**
**29**	**Eu fico preocupado com o que as outras pessoas pensam sobre o conteúdo da minha fala.**	**1**	**2**	**3**	**4**	**5**	**6**
**30**	**Eu me preocupo com a organização do conteúdo do que eu vou falar ao me apresentar para outras pessoas.**	**1**	**2**	**3**	**4**	**5**	**6**
**31**	**Eu me preocupo em causar uma boa impressão com o conteúdo da minha fala.**	**1**	**2**	**3**	**4**	**5**	**6**
**32**	**Antes de falar com outras pessoas, eu penso no conteúdo do que eu vou falar.**	**1**	**2**	**3**	**4**	**5**	**6**
**33**	**Eu me preocupo com o conteúdo do que eu vou falar quando eu sou convidado(a) a dar minhas opiniões.**	**1**	**2**	**3**	**4**	**5**	**6**
**34**	**Eu não fico preocupado(a) com o que as outras pessoas pensam sobre o conteúdo do que eu vou falar.**	**1**	**2**	**3**	**4**	**5**	**6**
**35**	**Eu fico preocupado(a) com o que as outras pessoas pensam sobre o movimento da minha face quando eu falo.**	**1**	**2**	**3**	**4**	**5**	**6**
**36**	**Eu fico preocupado(a) com o que as outras pessoas pensam sobre o movimento do meu corpo quando eu falo.**	**1**	**2**	**3**	**4**	**5**	**6**
**37**	**Eu me preocupo em causar uma boa impressão com o movimento da minha face quando eu falo.**	**1**	**2**	**3**	**4**	**5**	**6**
**38**	**Eu me preocupo com o movimento da minha face quando eu sou convidado(a) a dar minhas opiniões.**	**1**	**2**	**3**	**4**	**5**	**6**
**39**	**Eu não me preocupo em causar uma boa impressão com os movimentos da minha face quando eu falo.**	**1**	**2**	**3**	**4**	**5**	**6**
