# Supplementary material for: Cross-cultural adaptation of the Brazilian version of the Speech-Specific Reinvestment Scale – SSRS
Source: Codas. 2022 Jan 21;34(4):e20200385. doi: 10.1590/2317-1782/20212020385 (PMC9886101; doi:10.1590/2317-1782/20212020385)
Supplement: Translation

# Translation: Equivalência cultural da versão brasileira do protocolo Speech-Specific Reinvestment Scale– SSRS

**Authors:** Bruna Rainho Rocha, Andus Wing-Kuen Wong, Estella Pui-Man Ma, Mara Behlau

**Affiliations:** 1 Programa de Pós-graduação em Distúrbios da Comunicação Humana, Departamento de Fonoaudiologia, Universidade Federal de São Paulo – UNIFESP - São Paulo (SP), Brasil; 2 Nam Shan Psychology Laboratory, Department of Social and Behavioural Sciences, University of Hong Kong, Hong Kong, China; 3 Division of Speech and Hearing Sciences, University of Hong Kong, Hong Kong, China

**Keywords:** Voz, Estudos de Validação, Comunicação, Qualidade da Voz, Fonoaudiologia, Questionários, Estudos de Avaliação, Tradução (produto)

## Abstract

**Objetivo:**

Realizar a equivalência cultural da versão brasileira da escala SSRS, por meio de sua adaptação cultural e linguística.

**Método:**

Após tradução da SSRS para o português brasileiro e retro tradução para o inglês, os itens foram comparados com o instrumento original. As discrepâncias existentes foram modificadas por consenso por um comitê de fonoaudiólogos, resultando na Escala de Reinvestimento Específico na Fala – EREF. A EREF tem 39 questões e seis alternativas na chave de resposta: “discordo totalmente”, “discordo”, “discordo ligeiramente”, “concordo ligeiramente”, “concordo” e “concordo totalmente”. A pontuação é a soma das pontuações médias de cada subdimensão da escala, sendo que os itens negativos não entram na contabilidade ou exigem pontuação reversa. Para a equivalência cultural, a EREF foi aplicada em um total de 74 profissionais em exercício de atividade envolvendo comunicação com público, falantes do português brasileiro como primeira língua, com acréscimo da opção “não aplicável” na chave de respostas, para identificação de questões não compreendidas ou não apropriadas para a população alvo e cultura brasileira.

**Resultados:**

A escala foi inicialmente aplicada em 56 participantes. Treze encontraram dificuldade para o preenchimento de 27 questões. Após a adaptação da tradução das sentenças não compreendidas ou consideradas inapropriadas, a EREF modificada foi aplicada em mais 13 respondentes e não foram encontradas barreiras culturais e/ou conceituais.

**Conclusão:**

Foi verificada equivalência cultural entre a SSRS e sua versão traduzida para o português brasileiro, a EREF. As próximas etapas para validação da EREF para o Português Brasileiro serão realizadas com a conclusão desta fase.

## INTRODUÇÃO

A comunicação é fundamental em todas as esferas de relacionamento humano, seja nas relações pessoais, empresariais ou educacionais. O termo comunicar é proveniente do latim – communicare – que significa “tornar comum”^(1)^. Ou seja, a comunicação é eficiente quando o interlocutor consegue transmitir a mensagem desejada ao receptor, que a compreende integralmente. Essa mensagem pode ser codificada de diversas maneiras, sendo elas verbais – por meio da fala ou escrita – ou não-verbais – como, por exemplo, pelo uso de gestos e expressões faciais. Em um discurso oral, a linguagem verbal e a não-verbal estão sempre presentes, quer o interlocutor esteja ciente delas ou não e para uma boa comunicação, é importante que uma esteja em concordância com a outra, se completando e em coerência.

A comunicação sempre foi importante e tem se destacado cada vez mais, visto que as profissões que utilizam a comunicação como principal fator do desenvolvimento do seu trabalho estão aumentando em detrimento dos trabalhos manuais^(2)^. Já ao final do século XX, verificou-se que 62% da força de trabalho baseava seu sustento nas habilidades de comunicação - audição, voz, fala e linguagem – e os 38% que não utilizavam a comunicação para o trabalho – como agricultores e operários – necessitavam dela para adequada inserção na sociedade. Ou seja, a comunicação é fundamental para o crescimento profissional e pessoal e cada vez mais a sua competência é exigida na sociedade^(3)^.

Desenvolver as habilidades de comunicação e tornar a linguagem verbal e não-verbal em concordância demanda treino e conhecimento. Para poder auxiliar aqueles com dificuldade e tornar esse treino mais específico e direto, é importante avaliar as habilidades envolvidas na comunicação, inclusive com protocolos de autoavaliação.

Os protocolos de autoavaliação são considerados importantes ferramentas de mensuração do conhecimento do indivíduo sobre o impacto de sua condição – seja ela um problema ou algo a ser aprimorado – em suas relações sociais e profissionais^(4)^. Apesar de seu uso frequente, a maioria deles ainda é direcionada para questões de saúde, como os questionários de qualidade de vida. No Brasil, ainda há poucos instrumentos capazes de investigar a autoavaliação da comunicação de um indivíduo. Porém, tendo em vista a importância da comunicação, é imprescindível que esse panorama seja modificado.

A “Escala de Reinvestimento Específico na Fala - EREF” (*Speech-Specific Reinvestment Scale -* SSRS)^(5)^ é uma nova medida psicométrica que tem por objetivo quantificar a predisposição de alguém para exercer controle e monitoramento conscientes sobre a fala. Engloba questões não apenas do controle consciente sobre os movimentos da fala (por exemplo, movimento da língua, lábio e mandíbula), mas também o monitoramento consciente do conteúdo e da maneira de falar, assim como da movimentação facial e corporal para alcançar o objetivo final da comunicação, isto é, para permitir que o destinatário perceba e compreenda o discurso.

A escala foi desenvolvida com referência à Teoria de Reinvestimento^(6-8)^ sobre controle do motor dos membros do corpo. De acordo com essa teoria as habilidades motoras requerem dois tipos de processamento: o implícito e o explícito. O primeiro é benéfico para habilidades motoras bem praticadas, relativamente automatizadas, trabalhando com o conhecimento já pré-estabelecido e requer poucos recursos de memória de atenção e de trabalho. Por outro lado, o segundo é baseado em regras e requer consideráveis recursos de atenção e memória de trabalho.

As pessoas podem ser reinvestidoras altas ou baixas e, as que reinvestem de modo elevado, têm maior tendência de falhar em situações estressantes que envolvem habilidades complexas e vinculadas a regras^(6-8)^. Assim, ao elaborar a escala SSRS, os autores tinham como hipótese que o traço de personalidade de predisposição da consciência do controle e monitoramento da fala estaria relacionado negativamente à performance na fala conversacional. Os resultados apontaram para a confirmação da hipótese.

Além disso, os resultados validaram quatro subdimensões da SSRS, uma de controle e três de monitoramento da fala: 1. Subdimensão da autoconsciência do movimento da fala - ACMF, 2. Subdimensão da consciência pública da maneira de falar - CPMF, 3. Subdimensão da consciência pública do conteúdo da fala - CPCF, 4. Subdimensão da consciência pública da movimentação durante a fala – CPMF^(5)^. A escala possibilita a avaliação das subdimensões de forma distinta ou conjunta, o que a torna ainda mais robusta para examinar quaisquer efeitos variáveis sobre desempenho da fala, inclusive não-verbais, conforme sugerido pela literatura da Teoria do Reinvestimento^(9)^.

Visto que a escala *Speech-Specific Reinvestment Scale –* SSRS^(5)^ foi desenvolvida em inglês, para o uso em outras línguas deve ser traduzida e culturalmente adaptada de acordo com as regras internacionais do *Scientific Advisory Committee of Medical Outcome Trust* ^(10)^.

O objetivo deste estudo foi realizar a equivalência cultural da versão brasileira da escala SSRS, por meio de sua adaptação cultural e linguística.

É importante ressaltar que, por se tratar de uma escala nova, ainda não há repercussão de seu uso, embora seja de grande potencial. A adaptação brasileira é a primeira a ser realizada com o instrumento.

## MÉTODO

Após a autorização formal dos autores para a utilização do instrumento *Speech-Specific Reinvestment Scale -* SSRS)^(5)^, a pesquisa foi aprovada pelo Comitê de Ética em Pesquisa da Universidade Federal de São Paulo/CEP UNIFESP (parecer n^o^ 4.356.465 e CAAE: 36606720.0.0000.5505). Todos os participantes assinaram o Termo de Consentimento Livre e Esclarecido – TCLE.

Participaram profissionais adultos em exercício de atividade envolvendo comunicação oral com público, com experiência mínima de três meses na posição. Não houve distinção de nível de uso da comunicação, idade ou nível socioeconômico-cultural para este estudo.

A versão original foi traduzida para o português brasileiro por duas fonoaudiólogas fluentes na língua estrangeira (tradutora 1 e tradutora 2). Essas traduções foram sobrepostas, resultando na primeira versão em português – VP, mantendo-se a integridade conceitual dos itens. Foi realizada a retro tradução por uma terceira fonoaudióloga, também fluente em inglês, sem acesso à versão original do instrumento e aos objetivos do estudo.

A tradução e a retro tradução foram comparadas entre si e ao instrumento original. As discrepâncias existentes foram analisadas e discutidas por um comitê composto por cinco fonoaudiólogos especialistas em voz, com proficiência na língua inglesa e conhecimento do vocabulário específico para avaliação da comunicação. As mudanças necessárias foram realizadas por consenso resultando na versão 1 do Comitê – a versão de equivalência semântica e do idioma, intitulada Escala de Reinvestimento Específico da Fala – EREF.

A EREF seguiu o protocolo original, permanecendo com 39 questões e seis alternativas para marcação da frequência de ocorrência da situação descrita em: discordo totalmente (1), discordo (2), discordo ligeiramente (3), concordo ligeiramente (4), concordo (5), concordo totalmente (6). Mantiveram-se ainda, nesse momento, as quatro dimensões subdimensões da escala, sendo elas: 1. Subdimensão da autoconsciência do movimento da fala, 2. Subdimensão da Consciência pública da maneira de falar, 3. Subdimensão da Consciência pública do conteúdo da fala, 4. Subdimensão da Consciência pública da movimentação durante a fala. A pontuação de cada subdimensão do SSRS^(5)^ é calculada usando o método de pontuação média. A pontuação média composta da EREF é calculada pela soma igualmente ponderada das quatro pontuações médias das subdimensões. Para calcular as médias de cada subdimensão e pontuação média composta, você pode seguir as seguintes etapas:

Q.1-13: Subdimensão da autoconsciência do movimento da fala

Pontuação total: ___ ÷ 13 = Pontuação média da subdimensão: ___

Q.15-27: Subdimensão da consciência pública da maneira de falar

Pontuação total: ___ ÷ 13 = Pontuação média da subdimensão:___

Q.29-33: Subdimensão da consciência pública do conteúdo da fala

Pontuação total: ___ ÷ 5 = Pontuação média da subdimensão: ___

Q.35-38: Subdimensão da consciência pública da movimentação durante a fala

Pontuação total: ___ ÷ 4 = Pontuação média da subdimensão: ___

Total = Soma das pontuações médias:_________

É importante ressaltar ainda que existem quatro questões negativas na EREF - uma para cada subdimensão da escala. As questões 14, 28, 34 e 39 não fazem parte da pontuação total da EREF, pois foram desenvolvidas para checar a qualidade de respostas dos indivíduos, incluindo a confiabilidade. Caso o avaliador sinta a necessidade de inclui-las na pontuação, deve lembrar de realizar a pontuação reversa, como descrito no protocolo original^(5)^.

Para a equivalência cultural, a EREF foi aplicada em 74 profissionais da voz falada com o acréscimo da opção “não aplicável” para identificação de questões não compreendidas ou não apropriadas para a população alvo e cultura brasileira. Um espaço para o preenchimento de observações também foi acrescentado ao final da escala, permitindo que os voluntários explicassem suas dificuldades ou dúvidas. Caso os voluntários encontrassem alguma dificuldade para responder a escala, nova tradução e adaptação deveria ser realizada até que não se encontrassem mais barreiras culturais e/ou conceituais.

## RESULTADOS

Na primeira etapa 61 indivíduos responderam à EREF online. Cinco respostas estavam incompletas, portanto, foram incluídos 56 indivíduos na amostra. Destes, 13 assinalaram “não aplicável” em pelo menos uma das questões da escala. No total 27 questões foram identificadas como não compreendidas ou não apropriadas para a população alvo e cultura brasileira e foram discutidas pelo comitê de Fonoaudiólogos. A questão dois, por exemplo, foi alterada de “Eu estou ciente de como minha boca se move quando eu falo” para “Eu percebo como a minha boca se move quando eu falo”, a fim de utilizar uma linguagem de fácil acesso e mais próxima de todos os respondentes. Da mesma forma, optou-se por acrescentar o pronome “eu” em todas as questões, excluindo as frases com sujeitos ocultos para evitar qualquer erro de interpretação. A exemplo disso temos a questão cinco, na qual a frase “Eu penso no movimento dos meus lábios quando falo” foi ajustada para “Eu penso no movimento dos meus lábios quando eu falo”.

Além disso, o Comitê de Fonoaudiólogos analisou o que os respondentes haviam escrito no espaço para observações e foi possível concluir que muitos haviam assinalado “não aplicável” não porque as questões não haviam sido compreendidas e sim porque aqueles indivíduos nunca tinham se atentado ao contexto da questão. Ou seja, eles deveriam ter marcado “discordo”, pois não concordam com aquela afirmação em vez de “não aplicável”. Sendo assim, as instruções para o preenchimento da escala foram revistas para o preenchimento adequado.

A instrução inicial era: “Selecione as opções mais adequadas para indicar o quanto você concorda com cada uma das afirmações: (1) Discordo totalmente, (2) Discordo, (3) Discordo ligeiramente, (4) Concordo ligeiramente, (5) Concordo, (6) Concordo totalmente (Nota: Não há resposta certa ou errada para cada frase). Caso você não compreenda alguma das frases ou entenda que ela não é apropriada à escala, selecione a opção “não aplicável”. Ao final das frases, há um espaço para o preenchimento de observações.” Após a deliberação do Comitê de Fonoaudiólogos e o entendimento de que as instruções deveriam ser mais claras, houve alteração para: “Selecione as opções mais adequadas para indicar o quanto você concorda com cada uma das afirmações: (1) Discordo totalmente, (2) Discordo, (3) Discordo ligeiramente, (4) Concordo ligeiramente, (5) Concordo, (6) Concordo totalmente (Nota: Não há resposta certa ou errada para cada frase). Caso você não compreenda alguma das frases ou entenda que não faz sentido essa pergunta dentro do questionário, selecione a opção “não aplicável”. Essa marcação só deve ser realizada nesses casos. Se selecionar “não aplicável”, solicitamos que você explique suas dificuldades ou dúvidas no espaço dedicado para o preenchimento de observações ao final das frases.”

A EREF ajustada – versão 2 do Comitê de Fonoaudiólogos - foi aplicada em 13 novos respondentes, totalizando 69 voluntários incluídos na amostra e não foram identificadas barreiras culturais e/ou conceituais. O mapeamento dos participantes pode ser observado na Figura 1 e a caracterização deles no Quadro 1.

**Figura 1:** Organograma de preenchimento do questionário da pesquisa e inclusão e exclusão de sujeitos

**Quadro 1:** Caracterização da amostra de acordo com sexo, idade (em anos) e profissão

Variáveis	N	%
**Sexo**		
**Feminino**	**45**	**65,2**
**Masculino**	**24**	**34,8**
		
**Idade**		
**18** |–|**30**	**27**	**39,1**
**31** |–| **45**	**16**	**23,2**
**46** |–| **60**	**20**	**29**
**60** |–| **86**	**6**	**8,7**
		
**Profissão**		
**Professor(a)**	**9**	**13**
**Administrador(a)**	**6**	**8,7**
**Empreendedor(a)**	**6**	**8,7**
**Fonoaudiólogo(a)**	**5**	**7,2**
**Publicitário(a)**	**5**	**7,2**
**Engenheiro(a)**	**4**	**5,9**
**Advogado(a)**	**3**	**4,4**
**Aposentado(a)**	**3**	**4,4**
**Empresário(a)**	**3**	**4,4**
**Estudante**	**3**	**4,4**
**Analista**	**2**	**2,9**
**Arquiteto(a)**	**2**	**2,9**
**Consultor(a)**	**2**	**2,9**
**Cozinheiro(a)**	**2**	**2,9**
**Gerente**	**2**	**2,9**
**Jornalista**	**2**	**2,9**
**Médico(a)**	**2**	**2,9**
**Relações Públicas**	**2**	**2,9**
**Terapeuta Ocupacional**	**2**	**2,9**
**Cantor(a)**	**1**	**1,4**
**Dentista**	**1**	**1,4**
**Escritor(a)**	**1**	**1,4**
**Promotor de Justiça**	**1**	**1,4**

Essa última versão aplicada, resultou então na versão final da EREF, seguindo a equivalência cultural e linguística. Todo o processo de tradução, equivalência semântica e cultural da SSRS para o português brasileiro encontra-se no Quadro 2.

**Quadro 2:** Processo de tradução e adaptação cultural da escala Speech-Specific Reinvestment Scale - SSRS para o português brasileiro

**Questões**	**Versão Original em inglês**	**Tradução 1 inglês-português**	**Tradução 2 inglês-português**	**Versão sobreposta (VP)**	**Retro-tradução da VP**	**Versão 1 do Comitê**	**Versão Final do Comitê**
1	I examine my mouth movement when I speak.	Eu **analiso** o movimento da boca quando falo.	Eu **avalio/examino meu** movimento de boca quando eu falo.	Eu avalio meu movimento da boca quando eu falo.	When speaking I monitor my mouth movement	Eu avalio movimento de boca quando eu falo.	Eu avalio **O** movimento **DA MINHA BOCA** quando eu falo.
2	I am aware of the way my mouth moves when I speak.	Eu estou ciente de como minha boca se move quando falo.	Estou ciente da como minha boca se move quando falo.	Eu estou ciente de como minha boca se move quando eu falo.	I am aware of how my mouth moves when speaking	Eu estou ciente de como minha boca se move quando eu falo.	Eu **PERCEBO** como **A** minha boca se move quando eu falo.
3	I take time to reflect on my mouth movement after speaking.	Eu dedico tempo para refletir sobre o movimento da minha boca depois de falar.	Eu dedico tempo para refletir sobre o movimento da minha boca depois de falar.	Eu dedico tempo para refletir sobre o movimento da minha boca depois de falar.	After speaking, I think about my mouth movement	Eu dedico tempo para refletir sobre o movimento da minha boca depois de falar.	Eu dedico tempo para refletir sobre o movimento da minha boca depois de falar.
4	I take time to think about my mouth movement before speaking.	Eu dedico tempo para pensar no movimento da minha boca antes de falar.	Eu dedico tempo para pensar no movimento da minha boca antes de falar.	Eu dedico tempo para pensar no movimento da minha boca antes de falar.	Before speaking, I think about my mouth movement	Eu dedico tempo para pensar no movimento da minha boca antes de falar.	Eu dedico tempo para pensar no movimento da minha boca antes de falar.
5	I think about the movement of my lips when I speak.	Eu penso no movimento dos meus lábios quando falo.	Eu penso no movimento dos meus lábios quando falo.	Eu penso no movimento dos meus lábios quando falo.	I think about my lips movements when speaking	Eu penso no movimento dos meus lábios quando falo.	Eu penso no movimento dos meus lábios quando **EU** falo.
6	I think about the movement of my tongue when I speak.	Eu penso no movimento da minha língua quando falo.	Eu penso no movimento da minha língua quando falo.	Eu penso no movimento da minha língua quando falo.	I think about my toungue movement when speaking	Eu penso no movimento da minha língua quando falo.	Eu penso no movimento da minha língua quando **EU** falo.
7	I think about the movement of my jaw when I speak.	Eu penso no movimento da minha mandíbula quando falo.	Eu penso no movimento da minha mandíbula quando falo.	Eu penso no movimento da minha mandíbula quando falo.	I think about my jaw movement when speaking	Eu penso no movimento da minha mandíbula quando falo.	Eu penso no movimento da minha mandíbula quando **EU** falo.
8	I think about the movement of my larynx when I speak.	Eu penso no movimento da minha laringe quando falo.	Eu penso no movimento da minha laringe quando eu falo.	Eu penso no movimento da minha laringe quando falo.	I think about my larynx movement when speaking	Eu penso no movimento da minha laringe quando falo.	Eu penso no movimento da minha laringe quando **EU** falo.
9	I try to figure out how my mouth movement generates pronunciation.	Eu tento descobrir como o movimento da minha boca gera a pronúncia **DAS PALAVRAS.**	Eu tento descobrir como o movimento da minha boca gera a pronúncia.	Eu tento descobrir como o movimento da minha boca gera a pronúncia das palavras.	I try figuring out how my mouth moments produce words	Eu tento descobrir como o movimento da minha boca gera a pronúncia das palavras.	Eu tento descobrir como o movimento da minha boca gera a pronúncia das palavras.
10	I rethink how my mouth movement has helped me speak smoothly.	Eu repenso em **COMO O MOVIMENTO DA MINHA BOCA ME AJUDOU A FALAR SEM DIFICULDADES.**	Eu repenso em como o movimento da minha boca me ajudou a falar **SUAVEMENTE/SEM DIFICULDADES.**	Eu repenso em como o movimento da minha boca me ajudou a falar sem dificuldades.	I overthink how my mouth movement helped me speak easily	Eu repenso em como o movimento da minha boca me ajudou a falar sem dificuldades.	Eu repenso em como o movimento da minha boca me ajudou a falar sem dificuldades.
11	I think about how my mouth movement has helped me speak smoothly.	Eu penso em como o movimento da minha boca me ajudou a falar sem dificuldades.	Penso em como o movimento da minha boca me ajudou a falar **SUAVEMENTE**/sem dificuldades.	Eu penso em como o movimento da minha boca me ajudou a falar sem dificuldades.	I think how my mouth movement helped me speak easily	Eu penso em como o movimento da minha boca me ajudou a falar sem dificuldades.	Eu penso em como o movimento da minha boca me ajudou a falar sem dificuldades.
12	I try to understand how my speech arises from mouth movement.	Eu tento entender como minha fala surge do movimento **DA MINHA** boca.	Eu tento entender como minha fala surge do movimento da boca.	Eu tento entender como minha fala surge do movimento da minha boca.	I try to understand how my speech is produced from my mouth movement	Eu tento entender como minha fala surge do movimento da minha boca.	Eu tento entender como minha fala surge do movimento da minha boca.
13	I am aware of how my mouth moves for what I want to speak.	**EU** estou ciente de como minha boca se move para o que eu quero falar.	Estou ciente de como minha boca se move para o que eu quero falar.	Eu estou ciente de como minha boca se move para o que eu quero falar.	I am aware of how my mouth moves when speaking what I want	Eu estou ciente de como minha boca se move para o que eu quero falar.	Eu **PERCEBO** como minha boca se move para o que eu quero falar.
14	I do not try to figure out how my mouth movement generates pronunciation.	**EU** não tento descobrir como o movimento da minha boca gera **A PRONÚNCIA DAS PALAVRAS**.	Não tento descobrir como o movimento da minha boca gera pronúncia.	Eu não tento descobrir como o movimento da minha boca gera a pronúncia das palavras.	I do not try figuring out how my mouth moments produce words	Eu não tento descobrir como o movimento da minha boca gera a pronúncia das palavras.	Eu não tento descobrir como o movimento da minha boca gera a pronúncia das palavras.
15	I care about the voice being used to present myself to others.	Eu me preocupo com a minha voz **QUANDO EU ME APRESENTO** aos outros.	Eu me preocupo **COM A VOZ SENDO USADA** para me apresentar aos outros.	Eu me preocupo com a voz que eu uso para para me apresentar aos outros.	My voice concerns me when I introduce myself	Eu me preocupo com a voz que eu uso para para me apresentar aos outros.	Eu me preocupo com a voz que eu uso para me apresentar aos outros.
16	Before I talk to others, I think about how my pronunciation is.	Antes de falar com outras pessoas, **EU** penso em como está minha pronúncia.	Antes de falar com outras pessoas, penso em como está a minha pronúncia.	Antes de falar com outras pessoas, eu penso em como está minha pronúncia.	Before speaking with other people I think about my pronunciation	Antes de falar com outras pessoas, eu penso em como está minha pronúncia.	Antes de falar com outras pessoas, eu penso como está minha pronúncia.
17	I care about my pronunciation when I am invited to express my views.	Eu me preocupo com a minha pronúncia quando sou convidado a expressar meus pontos de vista.	Eu me preocupo com a minha pronúncia quando sou convidado a expressar meus pontos de vista.	Eu me preocupo com a minha pronúncia quando sou convidado a dar minhas opiniões.	My pronunciation concerns me when I am invited to give opinions	Eu me preocupo com a minha pronúncia quando sou convidado a dar minhas opiniões.	Eu me preocupo com a minha pronúncia quando **EU** sou convidado a dar minhas opiniões.
18	I am concerned about what other people think about my volume of speech.	Eu fico preocupado com o que as outras pessoas pensam sobre o volume **DE MINHA VOZ.**	Estou preocupado com o que as outras pessoas pensam sobre o meu volume **DE FALA.**	Estou preocupado com o que as outras pessoas pensam sobre o volume da minha fala.	I am concern of what people think about my speech loudness	Estou preocupado com o que as outras pessoas pensam sobre o volume da minha fala.	**EU FICO PREOCUPADO(A)** com o que as outras pessoas pensam sobre o volume da minha fala.
19	I care about my volume of speech when I am presenting myself to others.	Eu me preocupo com o **VOLUME DE MINHA VOZ** quando eu **ME APRESENTO** a outras pessoas.	Eu me preocupo com o **MEU VOLUME DE FALA** quando **ESTOU ME APRESENTANDO** para os outros.	Eu me preocupo com o volume da minha fala quando estou me apresentando a outras pessoas.	My speech loudness concerns me when I introduce myself	Eu me preocupo com o volume da minha fala quando estou me apresentando a outras pessoas.	Eu me preocupo com o volume da minha fala quando **EU** estou me apresentando **PARA** outras pessoas.
20	I worry about making a good impression with my volume of speech.	Eu me preocupo em causar uma boa impressão com o **VOLUME DE MINHA VOZ.**	Eu me preocupo em causar uma boa impressão no meu **VOLUME DE FALA**.	Eu me preocupo em causar uma boa impressão no volume da minha fala.	I am concern with making a good impression on my speech loudness	Eu me preocupo em causar uma boa impressão no volume da minha fala.	Eu me preocupo em causar uma boa impressão no volume da minha fala.
21	Before I talk to others, I think about my volume of speech.	Antes de falar com outras pessoas, eu penso no **VOLUME DA MINHA VOZ.**	Antes de falar com outras pessoas, penso no meu **VOLUME DE FALA**.	Antes de falar com outras pessoas, eu penso no volume da minha fala.	Before speaking with other people I think about my speech loudness	Antes de falar com outras pessoas, eu penso no volume da minha fala.	Antes de falar com outras pessoas, eu penso no volume da minha fala.
22	I care about my volume of speech when I am invited to express my view.	Eu me preocupo com o **VOLUME DE MINHA VOZ** quando sou convidado a expressar minha opinião.	Eu me preocupo com meu **VOLUME DE FALA** quando sou convidado a expressar minha opinião.	Eu me preocupo com o volume da minha fala quando sou convidado a dar minha opinião.	I am concern with my speech loudness when I am asked to give my opinion	Eu me preocupo com o volume da minha fala quando sou convidado a dar minha opinião.	Eu me preocupo com o volume da minha fala quando **EU** sou convidado(a) a dar minha opinião.
23	I am concerned about what other people think about my speech rate.	**EU FICO PREOCUPADO** com o que as outras pessoas pensam sobre a minha velocidade de fala.	**ESTOU PREOCUPADO** com o que as outras pessoas pensam sobre a minha velocidade de fala.	Eu fico preocupado com o que as outras pessoas pensam sobre a velocidade da minha fala.	I am concern of what people think about my speech rate	Eu fico preocupado com o que as outras pessoas pensam sobre a velocidade da minha fala.	Eu fico preocupado(a) com o que as outras pessoas pensam sobre a velocidade da minha fala.
24	I care about my speech rate when I am presenting myself to others.	Eu me preocupo com a velocidade de minha fala quando estou me apresentando a outras pessoas.	Eu me preocupo com a minha velocidade de fala quando estou me apresentando para outras pessoas.	Eu me preocupo com a velocidade da minha fala quando estou me apresentando a outras pessoas.	My speech rate concerns me when I introduce myself	Eu me preocupo com a velocidade da minha fala quando estou me apresentando a outras pessoas.	Eu me preocupo com a velocidade da minha fala quando **EU** estou me apresentando **PARA** outras pessoas.
25	I worry about making a good impression with my speech rate.	Eu me preocupo em causar uma boa impressão com a **VELOCIDADE DE MINHA FALA**.	Eu me preocupo em causar uma boa impressão com **A MINHA VELOCIDADE DE FALA**.	Eu me preocupo em causar uma boa impressão com a velocidade da minha fala.	I am concern with making a good impression on my speech rate	Eu me preocupo em causar uma boa impressão com a velocidade da minha fala.	Eu me preocupo em causar uma boa impressão com a velocidade da minha fala.
26	Before I talk to others, I think about my speech rate.	Antes de falar com outras pessoas, eu penso na **VELOCIDADE DE MINHA FALA.**	Antes de falar com outras pessoas, eu penso **NA MINHA VELOCIDADE DE FALA.**	Antes de falar com outras pessoas, eu penso na velocidade da minha fala.	Before speaking with other people I think about my speech rate	Antes de falar com outras pessoas, eu penso na velocidade da minha fala.	Antes de falar com outras pessoas, eu penso na velocidade da minha fala.
27	I care about my speech rate when I am invited to express my view.	Eu me preocupo com a **VELOCIDADE DA MINHA FALA** quando sou chamado a expressar minha opinião.	Eu me preocupo com a **MINHA VELOCIDADE DE FALA** quando sou convidado a expressar minha opinião.	Eu me preocupo com a velocidade da minha fala quando sou convidado a dar minha opinião.	I am concern with my speech rate when I am asked to give my opinion	Eu me preocupo com a velocidade da minha fala quando sou convidado a dar minha opinião.	Eu me preocupo com a velocidade da minha fala quando **EU** sou convidado a dar minha opinião.
28	Before I talk to others, I do not think about how my pronunciation is.	Antes de falar com outras pessoas, eu não penso em como está minha pronúncia **DAS PALAVRAS**.	Antes de falar com outras pessoas, não penso em como está minha pronúncia.	Antes de falar com outras pessoas, eu não penso em como está minha pronúncia das palavras.	Before speaking with other people I do not think about my pronunciation	Antes de falar com outras pessoas, eu não penso em como está minha pronúncia das palavras.	Antes de falar com outras pessoas, eu não penso em como está minha pronúncia das palavras.
29	I am concerned about what other people think about my speech content.	**EU FICO PREOCUPADO** com o que as outras pessoas pensam **SOBRE O CONTEÚDO DE MINHA FALA**.	**ESTOU PREOCUPADO** com o que as outras pessoas pensam **SOBRE O MEU CONTEÚDO DE FALA.**	Eu fico preocupado com o que as outras pessoas pensam sobre o conteúdo da minha fala.	The content of my speech concerns me when talking with other people	Eu fico preocupado com o que as outras pessoas pensam sobre o conteúdo da minha fala.	Eu fico preocupado com o que as outras pessoas pensam sobre o conteúdo da minha fala.
30	I care about how the content is organized when presenting myself to others.	Eu me preocupo com a organização do **CONTEÚDO DO QUE VOU FALAR** ao me apresentar a outras pessoas.	Eu me preocupo com a organização do conteúdo ao me apresentar a outras pessoas.	Eu me preocupo com a organização do conteúdo do que vou falar ao me apresentar a outras pessoas.	The content organization concerns me when I introduce myself	Eu me preocupo com a organização do conteúdo do que vou falar ao me apresentar a outras pessoas.	Eu me preocupo com a organização do conteúdo do que **EU** vou falar ao me apresentar **PARA** outras pessoas.
31	I worry about making a good impression with my speech content.	Eu me preocupo em causar uma boa impressão com o **CONTEÚDO DE MINHA FALA.**	Eu me preocupo em causar uma boa impressão com o **MEU CONTEÚDO DE FALA**.	Eu me preocupo em causar uma boa impressão com o conteúdo da minha fala.	I am concern with making a good impression with my speech content	Eu me preocupo em causar uma boa impressão com o conteúdo da minha fala.	Eu me preocupo em causar uma boa impressão com o conteúdo da minha fala.
32	Before I talk to others, I think about how the content of my speech is.	Antes de falar com outras pessoas, eu penso no **CONTEÚDO DO QUE VOU FALAR.**	Antes de falar com outras pessoas, penso **EM COMO ESTÁ O CONTEÚDO DO MEU DISCURSO.**	Antes de falar com outras pessoas, eu penso no conteúdo do que eu vou falar.	Before speaking with other people I think about my speech content	Antes de falar com outras pessoas, eu penso no conteúdo do que eu vou falar.	Antes de falar com outras pessoas, eu penso no conteúdo do que eu vou falar.
33	I care about my speech content when I am invited to express my views.	Eu me preocupo com o **CONTEÚDO DO QUE VOU FALAR QUANDO SOU CONVIDADO A DAR MINHAS OPINIÕES.**	Eu me preocupo com o **CONTEÚDO DA MINHA FALA QUANDO SOU CONVIDADO A EXPRESSAR MINHAS OPINIÕES.**	Eu me preocupo com o conteúdo do que vou falar quando sou convidado a dar minhas opiniões.	I am concern with my speech content when I am asked to give my opinion	Eu me preocupo com o conteúdo do que vou falar quando sou convidado a dar minhas opiniões.	Eu me preocupo com o conteúdo do que **EU** vou falar quando **EU** sou convidado(a) a dar minhas opiniões.
34	I am not concerned about what other people think about my speech content.	**EU NÃO FICO PREOCUPADO** com o que as outras pessoas pensam sobre o **CONTEÚDO DO QUE VOU FALAR**.	**NÃO ESTOU PREOCUPADO** com o que as outras pessoas pensam sobre o **CONTEÚDO DA MINHA FALA.**	Eu não fico preocupado com o que as outras pessoas pensam sobre o conteúdo do que eu vou falar.	I am not concern with what people think about my speech content	Eu não fico preocupado com o que as outras pessoas pensam sobre o conteúdo do que eu vou falar.	Eu não fico preocupado(a) com o que as outras pessoas pensam sobre o conteúdo do que eu vou falar.
35	I am concerned about what other people think about my facial movement when I speak.	**EU FICO PREOCUPADO** com o que as outras pessoas pensam sobre o **MOVIMENTO DE MINHA FACE** quando falo.	**ESTOU PREOCUPADO** com o que as outras pessoas pensam sobre o meu **MOVIMENTO FACIAL** quando falo.	Eu fico preocupado com o que as outras pessoas pensam sobre o movimento da minha face quando falo.	What people think about my face movements concerns me when speaking	Eu fico preocupado com o que as outras pessoas pensam sobre o movimento da minha face quando falo.	Eu fico preocupado(a) com o que as outras pessoas pensam sobre o movimento da minha face quando **EU** falo.
36	I am concerned about what other people think about my body movement when I speak.	**EU FICO PREOCUPADO** com o que as outras pessoas pensam sobre o movimento do meu corpo quando falo.	**ESTOU PREOCUPADO** com o que as outras pessoas pensam sobre o movimento do meu corpo quando falo.	Eu fico preocupado com o que as outras pessoas pensam sobre o movimento do meu corpo quando falo.	What people think about my body movements concerns me when speaking	Eu fico preocupado com o que as outras pessoas pensam sobre o movimento do meu corpo quando falo.	Eu fico preocupado(a) com o que as outras pessoas pensam sobre o movimento do meu corpo quando **EU** falo.
37	I worry about making a good impression with my facial movement when I speak.	Eu me preocupo em causar uma boa impressão com o **MOVIMENTO DE MINHA FACE** quando falo.	Eu me preocupo em causar uma boa impressão com **A MINHA MOVIMENTAÇÃO FACIAL** quando falo.	Eu me preocupo em causar uma boa impressão com o movimento da minha face quando falo.	I am concern with making a good impression regarding my face movements when I speak	Eu me preocupo em causar uma boa impressão com o movimento da minha face quando falo.	Eu me preocupo em causar uma boa impressão com o movimento da minha face quando **EU** falo.
38	I care about my facial movement when I am invited to express my views.	Eu me preocupo com o **MOVIMENTO DE MINHA FACE** quando sou **CHAMADO** a expressar **MEUS PONTOS DE VISTA.**	Eu me preocupo com **A MINHA MOVIMENTAÇÃO FACIAL** quando sou **CONVIDADO** a expressar **MINHAS OPINIÕES.**	Eu me preocupo com o movimento da minha face quando sou convidado a dar minhas opiniões.	I am concern with my face movement when I am asked to give my opinion	Eu me preocupo com o movimento da minha face quando sou convidado a dar minhas opiniões.	Eu me preocupo com o movimento da minha face quando **EU** sou convidado(a) a dar minhas opiniões.
39	I do not worry about making a good impression with my facial movement when I speak.	Eu não me preocupo em causar uma boa impressão com os **MOVIMENTOS DE MINHA FACE** quando falo.	Não me preocupo em causar uma boa impressão com a minha **MOVIMENTAÇÃO FACIAL** quando falo.	Eu não me preocupo em causar uma boa impressão com os movimentos da minha face quando falo.	I am not concern with making a good impression regarding my face movements when I speak	Eu não me preocupo em causar uma boa impressão com os movimentos da minha face quando falo.	Eu não me preocupo em causar uma boa impressão com os movimentos da minha face quando **EU** falo.

Fonte: elaborada pelos autores

A composição final da versão brasileira traduzida e culturalmente adaptada do SSRS, chamada de EREF (Anexo 1), apresenta 39 itens, como o protocolo original.

## DISCUSSÃO

A comunicação desempenha um grande poder dentro da sociedade atual. Utilizando a comunicação, podemos convencer, persuadir, influenciar, despertar interesses e sentimentos, e ainda provocar expectativas no interlocutor^(11)^. Dentro de uma organização, por exemplo, a comunicação bem utilizada pode estabelecer relações pacíficas, homogeneização e integração de ideias. A comunicação é um meio e também uma ferramenta e tendo em vista o seu papel fundamental, é importante que exista como avaliá-la, em seus diferentes aspectos. Para essa finalidade, a SSRS foi criada.

Quando um novo protocolo é proposto, existem regras no seu desenvolvimento para que não haja dificuldades na sua aplicação com a população alvo. O mesmo deve ser pensado para a utilização deste protocolo em outros países e, por isso, a validação do protocolo para cada língua e cultura é essencial. A obtenção da equivalência cultural é a primeira etapa para a validação de protocolos e visa a eliminação de barreiras culturais e linguísticas entre o instrumento e sua população alvo em diferentes países^(10)^. O modelo de equivalência cultural utilizado para esta pesquisa já foi realizado com sucesso em validações de outros protocolos no Brasil^(12-15)^.

A equivalência cultural da SSRS iniciou-se com a adaptação cultural e linguística, na qual é aplicada a versão da escala para a equivalência semântica e do idioma. Nessa fase, 27 questões foram identificadas como não compreendidas ou não apropriadas para a população alvo e cultura brasileira e foram discutidas pelo comitê de Fonoaudiólogos. O alto número de questões com barreiras para a população alvo (69%) levantou a discussão por parte do Comitê de Fonoaudiólogos a respeito do entendimento da escala por parte da população alvo. Foi observado que muitos respondentes não haviam compreendido a real função da opção “não aplicável” e em vez de assinalá-la quando acreditavam que a questão não era adequada à escala, alguns estavam assinalando essa opção por não realizarem o contexto da questão em suas tarefas de comunicação. Ou seja, em vez de responder à questão seguindo a frequência de respostas de 1 a 6, os respondentes assinalavam “não aplicável”. Essa situação levou à necessidade de rever as instruções para preenchimento da escala e, em nova aplicação, não foram observadas dificuldades.

A etapa de adaptação cultural é essencial para que a linguagem da escala se aproxime da população alvo e para que a consistência da forma de comunicação seja mantida em todas as questões. Essa etapa deve ser realizada quantas vezes for preciso até que a escala/o protocolo seja aceito pela população alvo. A SRSS para o português brasileiro precisou de duas revisões para que fosse aceita.

Com a conclusão do processo de equivalência cultural e linguística para o Português Brasileiro, terá início o processo de validação da EREF, nome da escala adaptada. Seu uso será importante, visto que a maioria dos protocolos de autoavaliação de voz é direcionada para doenças específicas, não são adequados para indivíduos com vozes saudáveis e não contemplam outras questões de comunicação oral. A SSRS é uma escala específica para profissionais da voz e será fundamental no Brasil para avaliar quem utiliza a voz como seu instrumento de trabalho independentemente do nível de classificação do uso profissional da voz.

## CONCLUSÃO

Foi verificada equivalência cultural entre a SSRS e a sua versão traduzida para o português brasileiro, a EREF. Sendo assim, será dado início às outras de validação da EREF para o português brasileiro.

## Anexo 1. **Versão traduzida e culturalmente adaptada da escala *Speech-Specific Reinvestment Scale –* SSRS, chamada Escala de Reinvestimento Específico da Fala – EREF**

Pergunta 1 a 39: As frases a seguir descrevem sua autorreflexão enquanto fala no português brasileiro.

As questões precisam ser lidas com atenção. Algumas são positivas e outras negativas.

Selecione as opções mais adequadas para indicar o quanto você concorda com cada uma das questões:

1: Discordo totalmente

2: Discordo

3: Discordo ligeiramente

4: Concordo ligeiramente

5: Concordo

6: Concordo totalmente

(Nota: Não há resposta certa ou errada para cada frase)

**Table:**

		**Discordo Totalmente**			**Concordo Totalmente**		
**1**	**Eu avalio o movimento da minha boca quando eu falo.**	**1**	**2**	**3**	**4**	**5**	**6**
**2**	**Eu percebo como a minha boca se move quando eu falo.**	**1**	**2**	**3**	**4**	**5**	**6**
**3**	**Eu dedico tempo para refletir sobre o movimento da minha boca depois de falar.**	**1**	**2**	**3**	**4**	**5**	**6**
**4**	**Eu dedico tempo para pensar no movimento da minha boca antes de falar.**	**1**	**2**	**3**	**4**	**5**	**6**
**5**	**Eu penso no movimento dos meus lábios quando eu falo.**	**1**	**2**	**3**	**4**	**5**	**6**
**6**	**Eu penso no movimento da minha língua quando eu falo.**	**1**	**2**	**3**	**4**	**5**	**6**
**7**	**Eu penso no movimento da minha mandíbula quando eu falo.**	**1**	**2**	**3**	**4**	**5**	**6**
**8**	**Eu penso no movimento da minha laringe quando eu falo.**	**1**	**2**	**3**	**4**	**5**	**6**
**9**	**Eu tento descobrir como o movimento da minha boca gera a pronúncia das palavras.**	**1**	**2**	**3**	**4**	**5**	**6**
**10**	**Eu repenso em como o movimento da minha boca me ajudou a falar sem dificuldades.**	**1**	**2**	**3**	**4**	**5**	**6**
**11**	**Eu penso em como o movimento da minha boca me ajudou a falar sem dificuldades.**	**1**	**2**	**3**	**4**	**5**	**6**
**12**	**Eu tento entender como minha fala surge do movimento da minha boca.**	**1**	**2**	**3**	**4**	**5**	**6**
**13**	**Eu percebo como minha boca se move para o que eu quero falar.**	**1**	**2**	**3**	**4**	**5**	**6**
**14**	**Eu não tento descobrir como o movimento da minha boca gera a pronúncia das palavras.**	**1**	**2**	**3**	**4**	**5**	**6**
**15**	**Eu me preocupo com a voz que eu uso para me apresentar aos outros.**	**1**	**2**	**3**	**4**	**5**	**6**
**16**	**Antes de falar com outras pessoas, eu penso como está minha pronúncia.**	**1**	**2**	**3**	**4**	**5**	**6**
**17**	**Eu me preocupo com a minha pronúncia quando eu sou convidado a dar minhas opiniões.**	**1**	**2**	**3**	**4**	**5**	**6**
**18**	**Eu fico preocupado(a) com o que as outras pessoas pensam sobre o volume da minha fala.**	**1**	**2**	**3**	**4**	**5**	**6**
**19**	**Eu me preocupo com o volume da minha fala quando eu estou me apresentando para outras pessoas.**	**1**	**2**	**3**	**4**	**5**	**6**
**20**	**Eu me preocupo em causar uma boa impressão no volume da minha fala.**	**1**	**2**	**3**	**4**	**5**	**6**
**21**	**Antes de falar com outras pessoas, eu penso no volume da minha fala.**	**1**	**2**	**3**	**4**	**5**	**6**
**22**	**Eu me preocupo com o volume da minha fala quando eu sou convidado(a) a dar minha opinião.**	**1**	**2**	**3**	**4**	**5**	**6**
**23**	**Eu fico preocupado(a) com o que as outras pessoas pensam sobre a velocidade da minha fala.**	**1**	**2**	**3**	**4**	**5**	**6**
**24**	**Eu me preocupo com a velocidade da minha fala quando eu estou me apresentando para outras pessoas.**	**1**	**2**	**3**	**4**	**5**	**6**
**25**	**Eu me preocupo em causar uma boa impressão com a velocidade da minha fala.**	**1**	**2**	**3**	**4**	**5**	**6**
**26**	**Antes de falar com outras pessoas, eu penso na velocidade da minha fala.**	**1**	**2**	**3**	**4**	**5**	**6**
**27**	**Eu me preocupo com a velocidade da minha fala quando eu sou convidado a dar minha opinião.**	**1**	**2**	**3**	**4**	**5**	**6**
**28**	**Antes de falar com outras pessoas, eu não penso em como está minha pronúncia das palavras.**	**1**	**2**	**3**	**4**	**5**	**6**
**29**	**Eu fico preocupado com o que as outras pessoas pensam sobre o conteúdo da minha fala.**	**1**	**2**	**3**	**4**	**5**	**6**
**30**	**Eu me preocupo com a organização do conteúdo do que eu vou falar ao me apresentar para outras pessoas.**	**1**	**2**	**3**	**4**	**5**	**6**
**31**	**Eu me preocupo em causar uma boa impressão com o conteúdo da minha fala.**	**1**	**2**	**3**	**4**	**5**	**6**
**32**	**Antes de falar com outras pessoas, eu penso no conteúdo do que eu vou falar.**	**1**	**2**	**3**	**4**	**5**	**6**
**33**	**Eu me preocupo com o conteúdo do que eu vou falar quando eu sou convidado(a) a dar minhas opiniões.**	**1**	**2**	**3**	**4**	**5**	**6**
**34**	**Eu não fico preocupado(a) com o que as outras pessoas pensam sobre o conteúdo do que eu vou falar.**	**1**	**2**	**3**	**4**	**5**	**6**
**35**	**Eu fico preocupado(a) com o que as outras pessoas pensam sobre o movimento da minha face quando eu falo.**	**1**	**2**	**3**	**4**	**5**	**6**
**36**	**Eu fico preocupado(a) com o que as outras pessoas pensam sobre o movimento do meu corpo quando eu falo.**	**1**	**2**	**3**	**4**	**5**	**6**
**37**	**Eu me preocupo em causar uma boa impressão com o movimento da minha face quando eu falo.**	**1**	**2**	**3**	**4**	**5**	**6**
**38**	**Eu me preocupo com o movimento da minha face quando eu sou convidado(a) a dar minhas opiniões.**	**1**	**2**	**3**	**4**	**5**	**6**
**39**	**Eu não me preocupo em causar uma boa impressão com os movimentos da minha face quando eu falo.**	**1**	**2**	**3**	**4**	**5**	**6**
